# BeGraft Aortic Stents: A European Multi-Centre Experience Reporting Acute Safety and Efficacy Outcomes for the Treatment of Vessel Stenosis in Congenital Heart Diseases

**DOI:** 10.3390/jcdd11070192

**Published:** 2024-06-25

**Authors:** Micol Rebonato, Mara Pilati, Sophie Malekzadeh Milani, Damien Bonnet, Emma Pascall, Matthew Jones, Pedro Betrian, Lisa Bianco, Hugues Lucron, Sebastien Hascoet, Alban-Elouen Baruteau, Luca Giugno, Gianfranco Butera

**Affiliations:** 1 Department of Pediatric Cardiology, Cardiac Surgery and Heart Lung Transplantation, Bambino Gesù Children’s Hospital, IRCCS, 00165 Rome, Italy; mara.pilati@opbg.net (M.P.); gianfranco.butera@opbg.net (G.B.); 2 Centre de Référence Malformations Cardiaques Congénitales Complexes—M3C, Hôpital Universitaire Necker Enfants Malades, F-75015 Paris, France; guiti.milani@gmail.com (S.M.M.); damien.bonnet1@gmail.com (D.B.); 3 Guy’s and St Thomas’ NHS Foundation Trust, Great Maze Pond, London SE1 9RT, UK; e.pascall@rbht.nhs.uk (E.P.); matthew.jones1@gstt.nhs.uk (M.J.); 4 Hospital Universitari Vall d’Hebron, 08035 Barcelona, Spain; pedro.betrian@vallhebron.cat (P.B.); l.bianco@rbht.nhs.uk (L.B.); 5 Hôpital Marie-Lannelongue, F-92350 Le Plessis-Robinson, France; hlucron@ghpsj.fr (H.L.);; 6 Groupe Hospitalier Paris Saint Joseph, F-75014 Paris, France; 7 Department of Pediatric Cardiology and Pediatric Cardiac Surgery, CHU Nantes, Nantes Université, FHU PRECICARE, F-44000 Nantes, France; albanelouen.baruteau@chu-nantes.fr; 8 CIC FEA 1413, INSERM, CHU Nantes, Nantes Université, F-44000 Nantes, France; 9 L’Institut du Thorax, INSERM, CNRS, CHU Nantes, Nantes Université, F-44000 Nantes, France; 10 UMR 1280, PhAN, INRAE, Nantes Université, F-44000 Nantes, France; 11 IRCCS Policlinico, 20097 San Donato, Italy; luca.giugno@grupposandonato.it

**Keywords:** BeGraft stent, congenital heart disease, vessel stenosis

## Abstract

Background: Stent implantation has become the preferred method of treatment for treating vessel stenosis in congenital heart diseases. The availability of covered stents may decrease complications and have an important role in the management of patients with complex anatomy. Aim: This study aims to evaluate the feasibility and safety of the pre-mounted cobalt–chromium stent-graft-covered ePTFE Aortic BeGraft in a broad spectrum of vascular lesions. Methods: This is a multicenter retrospective results analysis of 107 implanted BeGraft stents between 2016 and 2022 in six different European centers. Results: One hundred and four patients with a mean age of thirteen years (range 1–70 years) and with the body weight of 56.5 kg (range 11–115 kg) underwent the BeGraft stent implantation. Stents were implanted in the following conditions: aortic coarctation (74 patients), RVOT dysfunction (12 patients), Fontan circulation (7 patients), and miscellaneous (11 subjects with complex CHD). All the stents were implanted successfully. The median stent diameter was 16 mm (range 7–24 mm), and the median length was 39 mm (range 19–49 mm). Major complications occurred in five subjects (4.7%). During a median follow-up of fourteen (1–70) months, stents’ re-dilatation was performed in five patients. Conclusions: The BeGraft stent can be used safely and effectively in a wide spectrum of congenital heart diseases. Whether these good results will be stable in the longer term still needs to be investigated in a follow-up given its recent introduction into clinical practice, in particular regarding stent fracture or neointimal proliferation.

## 1. Introduction

Stent implantation for the treatment of native vessel or prosthetic conduit stenosis in congenital heart diseases has become a valid method of therapy. Although covered stents were initially used as a rescue procedure for vascular complications, especially for aortic coarctation, they have recently been adopted as a first-line treatment option for various kinds of lesions. The covered stents are mostly preferred for patients with complex and atretic aortic coarctation, concomitant patent ductus arteriosus, high risk of aortic wall complications, aneurysm [1], and in miscellaneous conditions such as calcified conduit stenosis in pulmonary position prior to percutaneous valve implantation, in Fontan circuit obstruction, to close TCPC fenestration [2], or as bail-out procedures when a complication occurs. Availability of covered stents may decrease complications and play an important role in the management of patients with complex anatomy. The ideal stent for use in congenital heart disease should have some specific characteristics, such as expandability to a large diameter; provide stability and radial force; and have a lower profile in order to be also used in the pediatric population.

Until a few years ago, only a limited number of covered stents were available for congenital heart disease with published optimal results but with some concerns regarding the diameter obtained, the profile, and membrane stability [1,2]. In November 2016, a new pre-mounted stent graft covered with ePTFE tubing (the BeGraft Aortic, Bentley InnoMed, Hechingen, Germany) received CE-mark approval.

We now present a multi-institutional experience using the pre-mounted cobalt–chromium stent-graft-covered ePTFE Aortic BeGraft in a broad spectrum of vascular lesions in congenital heart disease.

## 2. Methods

### 2.1. Study Design, Study Groups, and Procedural Data

This is a multicenter retrospective analysis of all consecutive BeGraft Aortic stents implanted in CHD patients from October 2016 until July 2022. All data about patients, types of procedures, and technical characteristics of the stents (Table 1 and Table 2) were collected from seven different European centers: Ospedale Pediatrico Bambino Gesu’, Rome, Italy (19 patients); Evelina Children Hospital, London, UK (15 patients); San Donato, Milan, Italy (11 patients); CHU de Nantes, L’institut du thorax, Nantes, France (4 patients); Vall d’Hebron Hospital, Barcelona, Spain (14 patients); Université de Paris Cité, Hospital Necker-Enfants malades, Paris, France (27 patients); and Marie Lannelongue hospital, Le Plessis-Robinson, France (14 patients). Approval for the study was obtained from the Clinical Research Ethics Committee of Ospedale Pediatrico Bambino Gesu’, Rome, Italy. Written informed consent was signed by the patients or their legal administrator, allowing us to perform the procedure and to use their clinical records.

According to indication, patients were divided into 4 groups: (1) coarctation of the aorta (CoA), (2) RVOT stenting, (3) Fontan, and (4) miscellaneous group.

In Groups 1, 2, and 3, the efficacy of stent implantation was defined as a reduction of the pressure gradient above 50% from the baseline, a significative increase in the diameter of the stenotic vessel or the conduit more than 1/3 the initial diameter, measured immediately after the procedure. In the Fontan group, the effectiveness of the fenestration closure was measured by the increase in oxygen saturation.

### 2.2. Stent Characteristics

**Figure 1:**
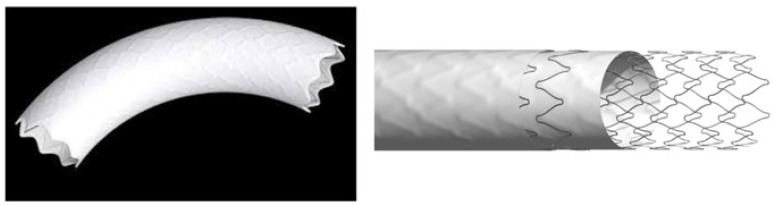
Begraft aortic stent.

The Bentley “BeGraft” Aortic Stent (Figure 1) is a hybrid open-cell stent design composed of L605 cobalt–chromium (CoCr) alloy, a synthetic polytetrafluoroethylene (ePTFE) graft with multiple micropores mounted on an expandable semi-compliant balloon, with platinum or iridium markers. It is available in three different stent designs (working diameters from 12 to 14 mm, 16 to 18, and 20 to 24 mm). The labeled stent graft length ranges from 19 to 58 mm, and the labeled expanded graft outer diameter ranges from 12 to 24 mm. Of particular importance at this stage, based on in vitro testing, this is the only stent that, in the version with the largest outer diameter, can be safely dilated up to 30 mm without jeopardizing the sleeve coverage.

### 2.3. Statistics

Continuous data are expressed as median (minimum–maximum range). Prevalence is reported as numbers and percentages.

## 3. Results

One hundred and seven consecutive Begraft stents were implanted in one hundred and four patients, male 58%, with a mean age of thirteen years (range 1–70) and with a mean body weight of 56.5 kg (range 11–115). Patients’ characteristics and procedural data are detailed in Table 1 and Table 2.

### 3.1. Coarctation Group

**Figure 2:**
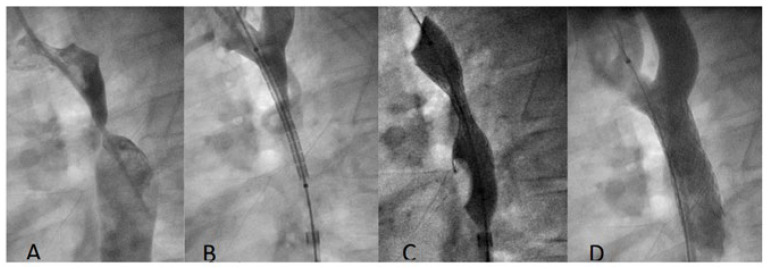
Coarctation treatment in 8-year-old boy. (**A**): Aortogram shows subatretic isthmic coarctation; (**B**): a 12 × 39 mm BeGraft stent is positioned through a 9 F sheath; (**C**): deployment of stent; (**D**): aortogram shows good final position of the stent.

Seventy-five Aortic Begraft stents were implanted in seventy-four patients with aortic coarctation. General characteristics are reported in Table 1. Reasons for implantation were native aortic coarctation in 49 pts and aortic re-coarctation in 25 pts (after surgery 11 pts, after prior stenting 12 pts, and after balloon angioplasty 2 pts). Among the native coarctation patients, four subjects had a functional interrupted aortic arch with a pinhole communication to the descending aorta (Figure 2).

The choice for covered stent implantation was prophylactic in almost all patients except one, in whom an aortic native dissection was detected at the time of the procedure and treated successfully with a BeGraft stent of 24 × 48 mm.

Arterial vascular access was obtained with an anatomical approach in 26 patients, echo guided in 40 patients and with a surgical cut-down in 8 subjects. Technical characteristics of the procedure are reported in Table 1. In 3 patients, post-dilatation of the stent was performed with bigger balloons in order to achieve the desired size. The diameter of the aorta at the coarctation site increased from a median of 7 (0–14) to 15 (11–25) mm. Acute complications occurred in four subjects (5.4%): one aortic dissection above the upper part of the proximal site of the stent, treated conservatively in a patient that previously underwent surgical coarctectomy and CP bare-metal stent implantation. One subject had cerebral air embolization without neurological sequelae, and one subject experienced femoral artery thrombosis that required surgical revascularization. Finally, one patient had a false aneurysm of the right femoral artery. The closure of the arterial access was obtained via manual compression in 25 pts, with percutaneous devices in 41 subjects and with surgical closure in 8 patients.

During a median follow-up of fourteen (1–70) months, stent re-dilatation was performed in 5 patients because of functional re-coarctation due to somatic growth in 3 patients and because of the lack of complete expansion of the stent at the nominal diameter at the first implant in 2 subjects.

The re-dilatation was successful in all five patients with no final clinically relevant fracture or dysfunction of the stent. The mean aortic diameter increased from 12 mm (9–14) to 18 mm (14–22) with a foreshortening percentage of less than 13%. The coarctation pressure gradient decreased to less than 10 mmHg in all patients after the procedure. Non-compliant balloons have been used in all cases; three of them were high-pressure balloons. One patient aged eight years with genetic diseases with multiple vessel abnormalities including a long-tract thoracic aortic coarctation showed a severe intrastent neo-intimal proliferation 36 months after the procedure that required surgical repair with extraanatomical bypass. Late complications occurred in two subjects: in one patient, the intrastent aneurysm was detected with an angio-CT 24 months after the procedure and was treated with a second covered CP stent. This event was probably related to a small rupture in the PTFE coating. Finally, an adult subject developed an aneurysm of the descending aorta 1 cm below the implanted the Begraft Aortic stent and was treated with an endovascular prosthesis (Zenith Alpha Thoracic Endovascular Graft 30–201, Cook, Bloomington, IN, USA).

### 3.2. RVOT Group

Fourteen Begraft Aortic stents were implanted in twelve patients with dysfunctional RVOT. All the stents were prophylactically deployed in stenotic and calcified conduits prior to transcatheter valve implantation. General characteristics are reported in Table 1. The baseline diagnosis included the tetralogy of Fallot (1 pt), pulmonary atresia (4 pts), the congenitally corrected transposition of the great arteries with pulmonary stenosis (2 pts), a double-outlet right ventricle (2 pts), transposition of great arteries (1 pt), Ross operation (1 pt), and truncus arteriosus (1 pt). The technical characteristics of the procedure are reported in Table 1. In two patients after the first stent implantation, the dissection of the conduit was detected, and a second Bentley aortic stent was implanted. In one patient, the stent completely sealed the flap, while in the other patient, other two bare-metal EV3 stents and one covered CP stent needed to be deployed for complete leak exclusion. In one patient, the balloon ruptured after full inflation. This event was dealt with percutaneous balloon retrieval. In all patients, successful PPVI was performed. At a median follow-up of twelve months (1–45), all the patients were doing fine, with normal prosthetic pulmonary valve function.

### 3.3. Fontan Group

**Figure 3:**
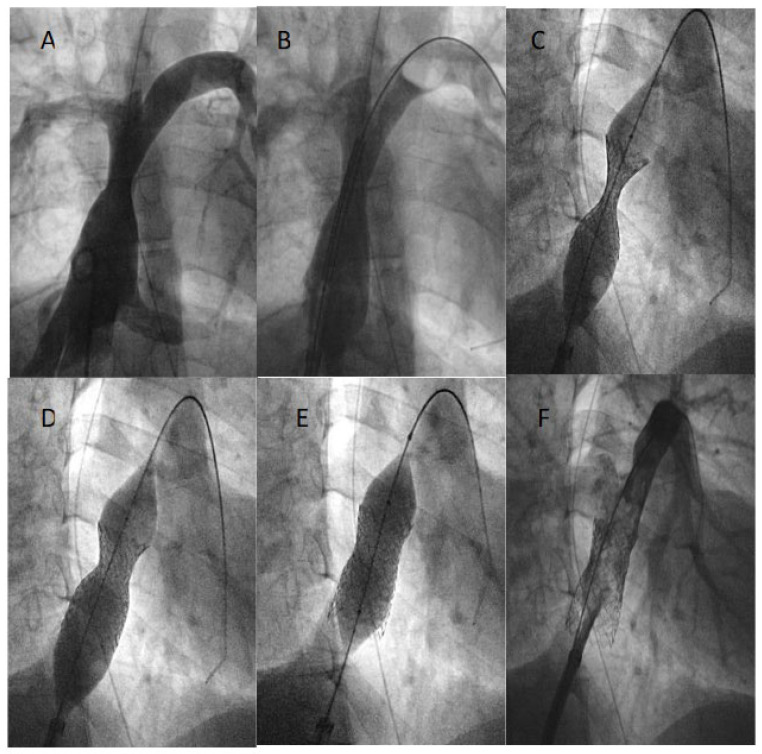
Restoration of Fontan conduit patency using a Begraft stent 22 × 48 mm (**A**–**D**). The stent is post dilated with a high-pressure balloon with good final result (**E**,**F**).

**Figure 4:**
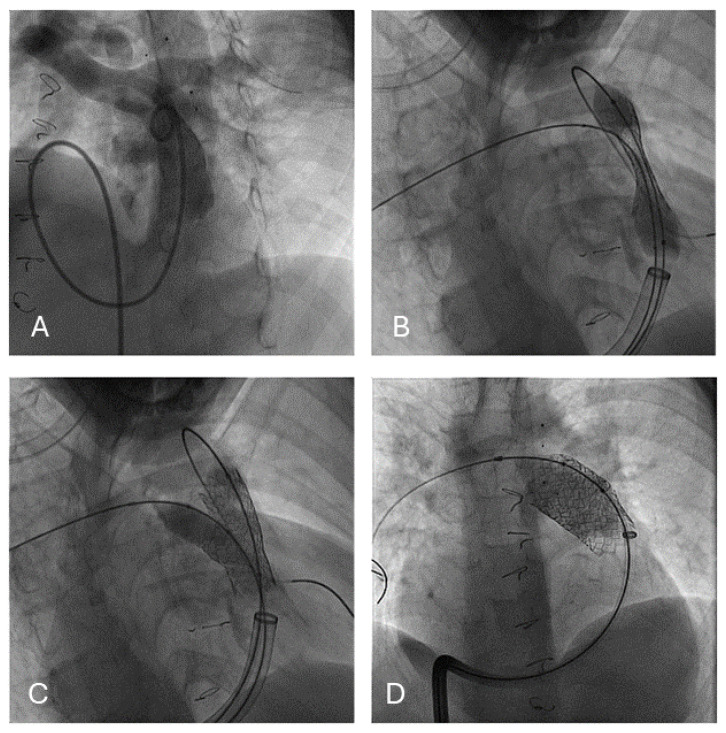
RVOT prestenting prior to pulmonary valve implantation using a Begraft stent 20 × 48 mm (**A**–**D**). Creation of a side branch hole for the right pulmonary artery with high pressure balloon (**C**).

Seven Begraft stents were implanted in seven patients with Fontan circulation for restoration of full Fontan conduit patency in three patients (Figure 3) and fenestration closure in four others. General and technical characteristics are reported in Table 1. The mean pressure gradient across the conduit was 4 mmHg (6-1) before the procedure and was completely abolished afterwards. In the fenestrated Fontan group, oxygen saturation increased from a median value of 86% (range 84–92%) to 96% (range 89–99%) after the procedure. In one patient, the balloon rupture occurred during stent inflation because of severe conduit calcification. A surgical femoral vein cut-down was needed to externalize the balloon and remove the catheter shaft from the groin. No further dilatation was needed during the median term follow-up of 14 (1–70) months. No residual shunt was detected across the fenestration (Figure 4).

### 3.4. Miscellaneus

Eleven patients with complex congenital heart disease underwent Begraft stent implantation. The indication for Begraft stenting was superior vena cava stenosis in three patients, rehabilitation of percutaneous Potts shunt in two patients, the exclusion of a portocaval fistula in one patient, relief of pulmonary arteries stenosis in three patients (two pts with univentricular physiology), and percutaneous PA debanding in two patients with ccTGA. General and technical characteristics are reported in Table 1.

No acute complications occurred. During the median follow-up of twenty-six months (1–70 months), one stent occlusion occurred due to thrombus formation with complete exclusion of the left pulmonary artery in a 4-year-old boy that was recruited with a left BT shunt. Two stents of 9 and 10 mm diameters in the Potts shunt position were successfully post-dilated with high-pressure balloons one year after the first procedure because of neo-intimal proliferation.

## 4. Discussion

Until a few years ago, covered stents were used mainly after aortic wall complications such as lesion recoil, vessel dissection or tear, and aneurysm formation. However, several reports showed the benefits of covered stents not only as a rescue therapy but also as a primary treatment for aortic coarctation, re-coarctation with or without aneurysm [3,4], stenting the right ventricular outflow tract prior to percutaneous valve implantation, particularly in stenotic and calcified conduits where the risk of rupture is high [5], and in miscellaneous conditions such as Fontan circuit obstruction or fenestration closure, pulmonary artery stenosis, and vein obstructions [6]. Butera G. [7] et al. described their initial experience in 2006 using CP-covered stents in patients with a variety of congenital heart diseases to treat patients with various kinds of stenosis and aneurysms of the aorta. However, the main disadvantage of the CP-covered stents is that they need quite large introducers in order to be delivered.

The characteristics of an ideal covered stent are a low profile, flexibility, good radial force, vascular conformability, adaptability to somatic growth, expansion without shortening and fracture resistance, and reliability and stability of the membrane.

Begraft Aortic stents have some of these technical advantages. They have a relatively low profile, come in a wide range of sizes and lengths, and have reasonable radial force because of CoCr alloy and mild foreshortening upon full expansion with a good membrane.

This multi-centric study describes the initial experience using covered BeGraft Aortic stents in the management of patients with simple and complex congenital heart disease. To our knowledge, this is the first report describing the utility of the Begraft Aortic stent in the treatment of a variety of vessel lesions even beyond aortic coarctation. Stent implantation was successful in all subjects where it was used.

In the literature, the vascular complication incidence after stent implantation is described to be about 2–5%. This complication is related to the size of arterial access, the sheath’s dimension, the patient’s weight, and the vascular access technique. Larger arterial sheaths and lower patient weight increase the risk of vascular complications [8]. In particular, the use of a covered CP stent requires a vascular sheath with a size of 3–4 Fr larger than the corresponding balloon. The COAST trial, which showed the effectiveness and safety of CP stent use in the treatment of aortic coarctation, reported an incidence of significant vascular complications of 3% [9]. The Bentley Begraft stent presents a very low profile as compared to other covered stents, and the sheath size is defined based on IFU indications. Among our population, the mean sheath dimension needed to access the vessel lesion was 12 Fr, and in the child population, the treatment of aortic coarctation was achieved using only a 9 Fr sheath through the femoral artery. In our study, only two vascular complications (1.9%) occurred: femoral artery thrombosis requiring surgical revascularization and femoral pseudoaneurysm. Both subjects had the stent delivered through a 14 Fr sheath. Previous reports about using Begraft Aortic stent showed the same results with low vascular complications incidence. The study of Yilmazer et al. [1], about utilizing the stent in eleven patients with aortic coarctation, described two vascular complications that were both conservatively treated. Balushi et al. [10] reported their experience with Begraft Aortic stent in five patients with near-atretic or severe coarctation without vascular complication.

In comparison with other stent grafts of large dimensions, the BeGraft Aortic stent is still rather flexible. This characteristic is related to the manufacture of the ePTFE covering and the CoCr alloy. The ePTFE tube in the Begraft stent is embedded in the stent and clamped between the first and the last stent struts, ensuring stability during deployment and safety during implantation and avoiding the risk of infolding of the covering membrane seen in other stents’ implantation. In the published experiences, tortuous and near-atretic coarctation were also treated with this graft successfully [10,11].

In our study, the BeGraft stent was used successfully, not only in aortic coarctation but also in RV to PA stenotic conduits and in pulmonary artery stenosis where flexibility is of particular importance. Successful implantation was achieved in all patients. The Begraft stents showed good grip and good stability in the RVOT position. No stent embolization occurred in our study, even in the RVOT group, where repetitive and laborious manipulations of extra-large sheaths had to be performed across the stent to facilitate the safe delivery of pulmonary valves. Two cases of calcific pulmonary conduit dissection were described in this series that required additional covered stent implantation to seal the leak. There are several stents on the market belonging to the group of large and extra-large stents and each type has certain advantages and disadvantages. Implanting the Begraft stent can create a safe and reliable valvular landing zone and provide a valid anchoring support for subsequent PPVI.

Furthermore, compared to other covered stents, the Begraft Aortic stents show less foreshortening, ranging from 8% to 25% in different studies [12].

Bentley stents were not only designed to match flexibility, resistance to fracture, and anchoring properties, but also high radial strength for stenosis relief. In our study, patients with aortic coarctation re-dilatation of the stent during the same procedure was performed in only three patients (2.8%) with high-pressure balloons in order to achieve the final expected aortic dimension. In the RVOT group and in the Fontan group, the final stent diameter was achieved in all the patients without further dilatation or other stent positioning. There was no need for secondary stenting in the case of important recoil or a residual gradient.

Only one patient treated with a Begraft Aortic stent for pulmonary stenosis experienced a late complete occlusion of the pulmonary artery due to thrombus formation, and the vessel was recruited with a BT shunt. Because of functional re-coarctation during follow-up, seven patients underwent successful stent re-dilatation: 5 pts in the coarctation group and 2 pts after pulmonary artery stenting. Only one patient, aged 8 years showed important aortic intrastent restenosis, necessitating a surgical extraanatomical bypass, but the patient had a genetic syndrome with multivessel abnormalities, including a long-tract coarctation segment.

These results are encouraging for the use of the Begraft Aortic stent in the group of patients who have not yet achieved final somatic growth because re-dilation can be performed with the larger balloons so that the stent can reach the final diameter of the thoracic aorta. In theory, these stents, like all other covered stents, can be re-dilatated for their entire duration in the vessel.

Vanagt WY et al. reported an incidence of re-dilatation of 24% in fifty-one patients with aortic coarctation treated with Cheatham Platinum Stent [2]. Yilmazer MM et al. [1] reported no need for the re-dilatation in eleven patients with aortic coarctation treated with the BeGraft Aortic stent at a median follow-up of fourteen months. The same experiment was described in the study of Al Balushi et al. Promphan et al. [10,11] reported a successful re-dilatation in two of twelve patients who underwent the BeGraft stent implantation for aortic coarctation treatment at a mean follow-up of ten months. In the same study, a second BeGraft stent implantation was reported during follow-up for a pseudoaneurysm formation caused by inadvertent distortion of the stent struts during stent implantation, causing damage to the PTFE patch.

In our study, one intrastent pseudoaneurysm was detected with a routine follow-up CT scan 24 months after a Begraft stent implantation in a near-atretic aortic coarctation and was treated with a covered Cheatham-Platinum Stent. Another patient experienced an aneurysm of the descending aorta 1 cm below the implanted BeGraft Aortic stent and was treated with endovascular graft prosthesis. Although no previous reports have been issued specifically on aneurysm/pseudoaneurysm formation after covered stent placement, a few reports enumerate pseudoaneurysm formation despite using covered CP stents. Pedra et al. [13] used covered CP stents and self-expandable stent grafts in 9 patients. The procedure was successful in all subjects. However, these authors reported the development of wall aneurysms in two patients despite using covered stents. In the study of Stassen J [14], describing the use of covered Cheatham Platinum stent in 78 patients with aortic coarctation, 2 patients experienced new aneurysm formation. Authors stated that despite an early and successful percutaneous repair, patients with coarctation will still develop aneurysm formation during long-term follow-up due to associated abnormal vascular phenotype, recommending strict long-term follow-up in these kinds of patients.

In our study, no stent migration was reported; however, we experienced two balloon ruptures during withdrawal after stent positioning: one in the RVOT group and the other in the Fontan group. Both patients had severely calcified conduits, and the presence of calcium leaf makes balloon deflation and retrieval more challenging. The disadvantage of this stent is the difficulty in separating the balloon from the stent during retrieval, and the forceful withdrawal can cause strut distortion with consequent aneurysm formation or embolization. Careful deflation of the balloon during the retrieval of the system is mandatory.

Finally, it must be noticed that balloon inflation has peculiar behaviors. In fact, during balloon inflation, the Begraft stent starts to expand in a completely unique way from both ends most of the time, with the distal part fully opened while the central part is only partially expanded in order to better center the lesion.

## 5. Conclusions

We reported a large experiment with BeGraft Aortic stents. Our results demonstrated that the BeGraft Aortic stent can be used safely and effectively in a wide spectrum of congenital heart diseases. The advantages of this stent are that it is deployed through a smaller sheath than other available devices because of its very low profile, a good radial force with good anchoring properties, good flexibility, and reliable membrane, both ends are completely covered with polytetrafluoroethylene and minimal foreshortening.

Whether these good results will be stable in a longer-term follow-up still needs to be investigated given the recent introduction of this practice into clinical practice in particular regarding stent fracture and neointimal proliferation.

## Figures and Tables

**Table 1:** Patients’ clinical and procedural characteristics.

	Total	Coarctation Group	Rvot Stenting	Fontan	Miscellaneous
Patients/stents n (%)	N = 104/N = 107	74 (71.1)/75 (70)	12 (11.5)/14 (13)	7 (6)/7 (6.5)	11 (10.5)/11 (10.2)
Age (years), median	13 (1–70)	26 (6–59)	19 (4–30)	15 (4–19)	11 (1–58)
Weight (kg), median (IQR)	56.5 (11–115)	58.5 (15–115)	58 (12–64)	57 (16–90)	58 (11–76)
Begraft stent size, median (IQR)	16 (7–24)	16 (7–24)	16 (7–24)	16 (14–22)	16 (7–24)
Begraft stent length, median (IQR)	39 (19–49)	39 (19–49)	39 (19–49)	39 (29–59)	39 (19–49)
Delivery sheath size (Fr), median	12 (9–16)	12 (9–14)	14 (9–16)	12 (9–14)	12 (9–14)
Fluoroscopy time (min), median	20 (6–68)	15 (6–60)	19 (8–56)	17 (7–48)	21 (9–68)

**Table 2:** Procedural data.

	Total	Coarctation Group	Rvot Stenting	Fontan	Miscellaneous
Patients	104	74	12	7	11
Implantation success, n (pt)	104	74	12	7	11
Serious procedural complications, n (%)	5 (4.8)	2 (2.7)	2 (16.6)	-	1 (9)
Minor procedural complication	4	2	1	1	-
Vascular access complications, n (%)	2	2	-	-	-
Delivery baloon rupture	2	-	1	1	-
Late stent redilatation	7	5	-	-	2
Follow-up (months), median (IQR)	14	14 (1–70)	12 (1–45)	14 (1–70)	14 (1–70)

## Data Availability

The original contributions presented in the study are included in the article, further inquiries can be directed to the corresponding author.
